# Brain Reward Pathway Dysfunction in Maternal Depression and Addiction: A Present and Future Transgenerational Risk

**DOI:** 10.17756/jrds.2015-017

**Published:** 2015-10-30

**Authors:** Benjamin C. Nephew, Christopher Murgatroyd, Florent Pittet, Marcelo Febo

**Affiliations:** 1 Department of Biomedical Sciences, Section of Neuroscience and Reproductive Biology, Tufts University Cummings School of Veterinary Medicine, North Grafton, MA, USA; 2 Manchester Metropolitan University School of Healthcare Science, Manchester, UK; 3 Department of Psychiatry, University of Florida College of Medicine, Gainesville, FL, USA

## Abstract

Two research areas that could benefit from a greater focus on the role of the reward pathway are maternal depression and maternal addiction. Both depression and addiction in mothers are mediated by deficiencies in the reward pathway and represent substantial risks to the health of offspring and future generations. This targeted review discusses maternal reward deficits in depressed and addicted mothers, neural, genetic, and epigenetic mechanisms, and the transgenerational transmission of these deficits from mother to offspring. Postpartum depression and drug use disorders may entail alterations in the reward pathway, particularly in striatal and prefrontal areas, which may affect maternal attachment to offspring and heighten the risk of transgenerational effects on the oxytocin and dopamine systems. Alterations may involve neural circuitry changes, genetic factors that impact monoaminergic neurotransmission, as well as growth factors such as BDNF and stress-associated signaling in the brain. Improved maternal reward-based preventative measures and treatments may be specifically effective for mothers and their offspring suffering from depression and/or addiction.

## Introduction

To coin the words of the British psychologist and psychiatrist John Bowlby, “what is believed to be essential for mental health is that the infant and young child should experience a warm, intimate and continuous relationship with his mother in which both find satisfaction and enjoyment” [1]. The critical role of maternal care and attachment on offspring development and the transgenerational transmission of factors influencing ‘survivability’ and genetic/epigenetic success cannot be understated. The challenges in understanding maternal care more deeply run along the same lines as the general challenges in broader fields of behavioral neuroscience, which is to determine specific regional patterns of neuronal activity, functional neural circuitry and genetic/epigenetic factors controlling maternal behavior and attachment. Alterations in epigenetic factors, which are likely to be extensive in response to internal and environmental challenges, and how these influence the gradual transmittal of stable behavioral patterns across generations is of substantial importance. Collectively, these separate lines of research could converge to help comprehend deficits in maternal care of offspring, such as those associated with depression and drug use. We provide here a brief review of an extensive literature on these broad topics, with a focus on deficits in the maternal reward pathway. We also provide some comparisons between the clinical literature and basic preclinical work that have moved this area of research a step forward. The present review will thus offer a concise overview of select topics spanning maternal reward deficits in depressed mothers, potential genetic factors involved and transgenerational effects, and the potential role of reward system alterations in maternal depression and drug use disorders.

## Maternal Reward Deficits in Depressed Mothers

Although it is not typically included in discussions of reward deficiency syndrome, maternal behavior is one of the most robust reward-mediated mammalian behaviors. This section will include discussion of studies on depression around the time of birth (peripartum depression, PPD) as well as during childhood (maternal depression or postnatal depression, PND), with a focus on postnatal depression and reward-mediated changes in maternal care. As discussed by Tom Insel, social attachment has much in common with addictive disorders through the involvement of the reward pathway [2]. Maternal rats presented with pups show increases neural activity in the nucleus accumbens (NAc) [3] and also show enhanced extracellular concentrations of dopamine in the Ventral Tegmental Area (VTA) [4]. Furthermore, lesions to the VTA impair pup directed behaviors [5, 6]. Using operant condition to determine the motivation to lever press for pups, it was shown that the rewarding effect of pups increases as maternal behavior develops [7]. Studies comparing the rewarding value of cocaine and pups indicate that a pup stimulus during early lactation is a more potent reward than cocaine [8, 9]. Based on this substantial evidence supporting the notion that in maternal rats pups are highly rewarding, deficiencies in maternal attachment behaviors may indicate adverse neuroadaptations in neuronal reward circuitry.

Appropriate maternal care involves active and timely emotional responding to infant cues and the regulation of these responses. While depression is partly associated with impaired serotonergic (5HT) pathways, it is also mediated by alterations in the mesolimbic dopamine system that can result in deficits in processing of rewards [10], which is critical for maternal care. Key reward related brain regions postulated to be involved in establishing the appropriate balance between maternal responses and regulation are the VTA, NAc) orbitofrontal cortex (OFC), prefrontal cortex (PFC), and striatum. Activation of these regions might establish infant stimuli as positive cues and enhance the motivation to express maternal care.

Behaviorally, depressed mothers are less likely to express sensitivity to infant related cues and instead display more hostile and intrusive behaviors towards their child [11, 12]. Several reports have documented impaired early parenting in depressed mothers [11, 13, 14], and postnatal major depression is associated with long term deficiencies in the maternal-infant bond [15–18]. Maternal attachment is dependent on the rewarding features of infant stimuli, and it has been postulated that differences in attachment styles are indicative of differences in the processing of sensory input. Adults that are able to successfully interpret infant-related affective information and predict future reward display secure patterns of attachment [19], where depressed mothers exhibit poor emotional coordination with their infants [20]. Depressed maternal-infant interaction often involves substantially lower levels of visual contact and smiling [12], and the NAc has been implicated in synchrony and intrusiveness in maternal care [21].

There is consistent evidence from imaging studies of healthy and depressed mothers that reward mediated responses to infant cues are altered in depressed mothers. Reward pathways are activated in response to infant cry in healthy mothers [22]. Mothers that deliver vaginally, which involves a substantial oxytocin (OXT) surge and enhanced bonding [23], display increased neural activation in the striatum compared to mothers who deliver by Cesarean-section [24]. Related work in sheep indicates that the reward pathway plays a critical role in the induction of central and peripheral OXT release during cervico-vaginal stimulation during parturition, and this interaction between parturition and reward pathway affects the onset of maternal care [25]. Healthy mothers display an increased neural response to the face of their own infant compared to an unknown infant in important reward related areas such as the VTA, ventral striatum, and PFC [26, 27], and the viewing of video of their own distressed infant increases activity in the substantia nigra and striatum of mothers [28]. In contrast, insecure mothers that lack a strong bond with their infant display decreased activation of the ventral striatum when viewing happy faces of their own infants. Overall, insecure mothers exhibit decreased activation in mesocorticolimbic pathways and increased activation in nigrostriatal pathways, including the PFC and insula [29, 30]. In support of the hypothesis that connections between the PFC and amygdala mediate secure attachment, women with PND have decreased neural activation in regions of the PFC [31] and also display decreased activity in the amygdala in response to positive visual stimuli from their own infants compared to non-depressed mothers [32]. Changes in both PFC and amygdala may mediate the impaired recognition of happy faces [33]. Depressed mothers show similar patterns of neural activity in response to the cries of their own and unfamiliar infants, and exhibit attenuated responses to their own infant cry in the OFC, NAc, and ventral striatum [34]. These data indicate that depressed mothers lack a specific neural response to the cry of their own infant, which may contribute to low levels of reward related maternal responses. Impaired preferences for their own infant combined with poor bond development may be mediated by neurobiological changes in the infants of depressed mothers who in turn exhibit deficits in face and voice preference [35]. The imaging studies suggest that treatments specifically aimed at increasing the reward response to infant cues may be effective at improving maternal care in depressed mothers and preventing the adverse transgenerational effects of PND on offspring. Given that a prior history of depression is the most reliable predictor of postpartum depression [36], more specific assessment of reward functioning prior to maternal periods may be used as a behavioral target to anticipate, prevent, and/or treat PPD. One potential target for potential preventative measures and treatments is the behaviorally active neuropeptide OXT.

The importance of OXT in affiliation and social bonding is well known and this neuropeptide plays a critical role in the expression of maternal behavior [37–39]. Connections between the medial preoptic area (mPOA), VTA, and NAc are involved in the rewarding aspects of social behaviors such as maternal care, and OXT acts in this combined maternal/reward circuit [2]. OXT has significant effects on the social reward pathway in rodent studies [40], potentially mediating deficient maternal care in depressed and anxious mothers who have been reported to have low OXT levels [41]. Breastfeeding increases OXT levels and elevates striatal activity when mothers hear the cry of their own infant [42]. Similar neural responses associated with postpartum OXT have been reported in suckling rodent dams [43]. In a chronic social stress (CSS) mediated rodent model of PPD [44, 45], OXT gene expression is reduced in the hypothalamus and amygdala of stress exposed rats that exhibit depressed maternal care, impaired lactation, and increased anxiety [46, 47], and OXT levels in the amygdala are correlated with maternal care [48]. A longitudinal study of 7000 mother-infant dyads revealed that breastfeeding is inversely correlated with the risk of maternal neglect [49] and this observation may involve OXT’s actions on the reward system. Clinical trials with intranasal OXT in depressed mothers have had mixed results [50], with acute OXT increasing general sadness but improving positive perceptions of the maternal-infant relationship [51]. A follow-up study looking at the effects of intranasal OXT on maternal protective responses to a stranger revealed enhanced protective responses [52]. Animal and human studies of OXT consistently and clearly indicate that it mediates multiple facets of maternal care, possibly through interactions with the reward pathway.

Taken together, these studies support the hypothesis that treatments aimed at addressing maternal specific reward deficits in depressed mothers may improve the formation of the maternal infant bond and ongoing maternal care, but there is a need for further research to identify effective treatments and optimize them. In improving maternal care, treatments that enhance brain reward function may prevent the adverse transgenerational effects of perinatal depression on offspring. It is also possible that maternal reward based treatments may prove more effective at treating depressive symptoms in the mother, whether it is directly through a reduction of anhedonia and/or indirectly through the improvement of the maternal-infant bond and maternal interactions.

## Genetic Factors Potentially Involved in Reward Deficiency in Maternal Depression

Genetic factors have been implicated in the etiology of perinatal mood disorders, as suggested by familial, twin, and adoption studies. A variety of reward-related genetic variants have been identified including those associated with monoaminergic neurotransmission, the stress response (e.g., HPA axis), neurogenesis (e.g., brain derived neurotrophic factor) and OXT. Preliminary evidence suggests these genetic variants may confer vulnerability to depression during and after pregnancy as well [53]. Many of the genetic associations are complex due to the critical role of environmental triggers; for some time, a gene-environment interaction has been recognized in the pathophysiology of depression, and this may well be the case for PPD since the three main risk factors are a past history of depression or anxiety (which may involve genetic and environmental factors), exposure to a stressful life event and a lack of social support [36, 54, 55]. The serotonergic system, in particular, plays a crucial role in depression. The serotonin transporter (5-HTT) has a polymorphism (5-HTTLPR) in the promoter region of the gene consisting of two alleles of a 43 bp insertion/deletion designated as long (l) and short (s). The s-allele results in a decreased transcription of the 5-HTT gene, leading to increased levels of serotonin in the synaptic cleft [56]. Studies have associated the s-allele with higher levels of trait anxiety [57] and with selective attention to negative, threat-related stressors [58]. Caspi et al. [59] were the first to show an effect of gene and environment interaction in modulating the risk for developing depression. It was reported by this group that individuals with the s-allele exhibited more depressive symptoms, compared to homozygous l-alleles, if exposed to stressful experiences during life [59].

Two studies have linked 5-HTTLPR genotype to maternal sensitivity. Specifically, the s-allele is associated with more sensitive parenting [60]. Two further studies have investigated the relationship between this polymorphism and PPD. Sanjuan et al. [61] linked the l-allele to PPD and reported this association at 8 weeks postpartum, but not during the immediate postpartum period or 32 weeks later [61]. Doornbos et al. [62] showed a trend for carriers of the l-allele toward increased depression scores at 6 weeks postpartum, suggesting the importance of evaluation time point [62]. A polymorphism in the serotonin receptor 2A gene (HTR2A) mediated levels of depressive symptoms in children with nurturing mothers but not among those exposed to non-nurturing mothering, supporting the role of gene-environment interactions [63]. Interestingly, a pilot study on a very limited number of participants linked 3 distinct single nucleotide polymorphisms (SNPs) in this gene with postpartum depression [64].

Two isoforms of the gene encoding tryptophan hydroxylase (TPH1 and TPH2), a major biosynthetic enzyme for serotonin, have been linked to depression. A study of SNPs in TPH1 found associations with comorbid depression and anxiety in a population-based sample of postpartum Taiwanese women [65]. Two further studies have highlighted the importance of the TPH2 gene. Lin et al. [66] found a SNP present only in women with peripartum major depression and anxiety disorders and Fasching et al. [67] linked the promoter region of this gene with depression values during and 8 months after pregnancy [66, 67].

Finally, two further genes implicated in monoaminergic pathways, namely catechol-O-methyltransferase (COMT) and monoamine oxidase A (MAO-A) have also been associated with perinatal depression during stress exposure [62]. The COMT-VAL158Met allele polymorphism has been shown to be a significant - though generally not an independent - risk factor for PPD [62, 68, 69]. Along this line, Pinsonneault et al. [70] demonstrated that SNPs in MAO-A and COMT exhibited additive effects in the development of PPD [70].

Studies of the gene encoding brain derived neurotrophic factor (BDNF) have reported potential links between the Val/Met polymorphism and PPD. Comasco et al. [71] found a significant association between the Met allele and development of PPD symptoms at 6 weeks postpartum, even when controlling for prepartum and postpartum environmental risk factors. This was most evident in mothers delivering during autumn/winter. A further interaction of this polymorphism was found with the 5-HTTLPR s-allele [71], which is involved in maternal sensitivity [60]. Another study in 227 subjects found no difference in BDNF genotype between depressed and non-depressed women [72]. Interestingly, recent findings link lower maternal serum BDNF levels in early pregnancy with antepartum depression [73] and altered DNA methylation at the BDNF promoter IV in response to maternal depression during pregnancy [74].

While there are numerous studies on the role of the hypothalamic-pituitary-adrenal (HPA) axis in the transgenerational transmission of depression, many do not focus on maternal depression and functional changes in the maternal reward pathway. Along with the rs242939 SNP of the CRHR1 gene [75], HPA targets such as the glucocorticoid receptor (GR) polymorphism BclI have been linked to PPD. However, an association study by Schneider et al. [76] in a cohort of 361 women without further risk factors for depressive or anxiety disorders found no significant links in the genes FKBP5, NR3C1, and CRHR1 with depression symptoms during or after pregnancy [76]. Additional data linking the HPA alterations in depressed mothers and offspring with reward deficiencies may yield key insight into preventing the transmission of the depressed phenotype from mother-to-child. It is possible that reducing HPA reactivity to differing stimuli in mothers may decrease depressive symptoms, but possibly not improve the mother-infant bond or enhance maternal care. HPA axis instability appears to be a key factor in increasing susceptibility to stress-related maternal mood disorders. Also, elevated levels of placental CRH have recently been identified as a marker of risk for the development of PPD symptoms [77].

Both animal and human studies have demonstrated that OXT promotes the arousal of basic emotional systems in maternal care [50]. A polymorphism study by Mileva-Seitz et al. [78] at the OXT and OXTR genes in 187 mothers at six months postpartum found 2 SNPs in OXT significantly associated with maternal vocalizing to the infant [78]. These polymorphisms also interacted with the quality of care mothers experienced in early life to predict variation in maternal care and postpartum depression. However, postpartum depression did not mediate the gene-environment effects of OXT SNPs on maternal care. The same group also reported that one of the SNPs interacted with early life adversity to predict variation in breastfeeding duration and depression, as genetic variation in OXT rs2740210 and early adversity was associated with postpartum depression and breastfeeding duration [79]. In contrast, while the OXTR SNP rs237885 did not associate with maternal behavior, it did associate with pre-natal (but not post-natal) depression score [78]. This illustrates the importance of variation in OXT genes, both alone and in interaction with early environment, as predictors of individual differences in human mothering.

Increased focus on gender specific gene-gene interactions and additional longitudinal studies of women who do and do not develop PPD will provide valuable insight into PPD etiology and facilitate the independent identification of long-term genetic risk factors and additional factors which interact with environmental stressors. In this work, increased use of animal models, which include an environmental trigger, will be useful.

## Maternal Reward Deficits, Maternal Care, and the Transgenerational Transmission of Depression

In Goodman and Gotlib’s model proposed in their 1999 review [80], they present a developmental model of the transmission of psychopathology from depressed mothers to their offspring which includes 4 central mechanisms: genetic predisposition, dysfunctional neuroregulatory mechanisms, maladaptive maternal behavior, and a stressful environment. It is acknowledged that the first three mechanisms may all have substantial genetic components. In addition, a stressful environment can lead to genetic predisposition for future generations through epigenetic changes. All of these related mechanisms could involve an impaired reward pathway. While animal models are useful for targeting individual mechanisms and specific components of a mechanism, perhaps what is needed in future models is greater integration of all of these mechanisms to improve the transgenerational construct validity and enhance translational success to effective and safe preventative measures and treatments, similar to what has been proposed for clinical studies [81].

Previous assessments have estimated that 15 million children live with a depressed parent (2009), and stressful family environments, such as those with a depressed mother, are a common risk factor for adolescent depression [82]. Depression in mothers is often associated with the display of depression in female offspring [80, 83–85]. It is possible that neurobiological factors contributing to adverse effects on the maternal-offspring bond may be present early in life and these might be associated with a high genetic risk for depression. There have been several human imaging studies of girls with high familial risk for developing depression. Study subjects were scanned prior to diagnosis with a major depressive or bipolar disorder. Importantly, mothers of the study subjects had a history of diagnosed mood disorder. Across several major studies it was shown that specific regions involved in processing rewards and losses were impaired compared to age matched and equally healthy controls in which mother were unaffected by a mood disorder [86–90]. Thus, high risk girls showed altered insular, striatal and cingulate processing of reward gains and losses in comparison to controls [86]. Sad mood induction in girls aged 9–14 resulted in greater blood oxygenation level dependent (BOLD) activation in areas of the amygdala and ventrolateral prefrontal cortex [87]. Self-regulation of sad mood produced a weaker BOLD activation of dorsal anterior cingulate and dorsolateral prefrontal cortex in high risk compared to low risk girls [87]. These and other examples illustrate the early presence of functional changes that may contribute later on in life to the development of a mood disorder. It is possible that such early onset of neural mechanisms (in the absence of behavioral expression of these conditions) may also be a contributing factor for PPD. A unifying theme across these studies is the presence of reward deficits, which might associate brain reward regions with PPD. The areas seem to span prefrontal cortical areas, cingulate cortex, insular cortex, striatum and amygdala, which have been associated with maternal responding to infant stimuli. In addition to inherited genetic risk factors, it has been hypothesized that non-inherited factors, especially maternal care, has robust mediating effects on psychopathology in the offspring of depressed mothers [91, 92]. There is evidence that an impaired maternal-child relationship mediates the severity of depression [93] and externalizing issues in the offspring of depressed mothers [94]. Behaviorally, this transmission is likely to be mediated by the increased display of hostility and decreased display of warmth in depressed mothers [95]. Maladaptive maternal interactions may also involve antisocial behavior in the mother [92, 96].

While some PPD interventions are starting to include attempts at addressing deficits in maternal care, most interventions have focused on the depression in the parent rather than the adverse effects of depression on maternal care [97]. Although remission of maternal depression has been associated with improved mental health in children [97, 98], other reports have not found strong links between depression treatment or remission and improved mother-child relationships [99] or offspring mental health [100, 101]. A recent meta-analysis of the effects of maternal psychotherapy on child and parental functioning concluded that treatment had mild-to-moderate beneficial effects on offspring [102], with counseling having the most substantial effect [103]. These modest beneficial effects of psychotherapy on the child indicate that there is potential for improvement and that a greater focus on enhancing the maternal bond may be worthwhile. However, some attempts to prevent postnatal depression by targeting the maternal bond have been unsuccessful [104], underscoring the challenges and need for further research in this area. Research on the rewarding aspects of parenting, specifically on the functional roles of DA, OXT and other neurotransmitter systems (discussed below) and how these modulate parenting behaviors and child mental health, would clearly benefit from longitudinal studies.

Bond development and maternal care, particularly synchrony in the fine social communication between mother and offspring constitute early life environmental characteristics with critical consequences on the onset and development of the offspring social brain [105]. Interestingly, these characteristics are impaired in maternal postpartum depression, strongly affecting the social functioning of offspring, including their affiliative behavior and corresponding ability to develop attachments with partners and offspring. Because of the difficulty in developing studies assessing multigenerational consequences of maternal depression on offspring neurophysiology and maternal care in humans, animal models have been developed to address this topic. One of the most common procedures to assess the effects of maternal stress on manipulating the maternal-offspring bond and maternal behavior is the maternal separation paradigm. Studies demonstrated strong consequences of maternal separation on the development of depressive-like syndromes; notably associated with impaired mesolimbic system development [106–108].

Another rodent model that has attempted to integrate the mechanisms discussed by Goodman and Gotlib in an ethologically and translationally relevant design is the CSS paradigm introduced earlier [80]. This social stress paradigm in lactating rat dams models intergenerational consequences of depressed maternal care using an early social environment representative of early life stress endured by children of depressed mothers, including both depressed maternal care and social conflicts [85, 109]. Daily exposure of lactating F0 rat dams to the social stress of a novel male intruder depresses maternal care, impairs lactation, increases maternal anxiety [44, 45, 48], and has robust adverse effects on the maternal care of F1 female offspring [46, 47, 110] and the social behavior of male and female F2 offspring [111]. Both F0 dams stressed by a male nest intruder and F1 dams exposed to this form of early life stress exhibit depressed maternal care, impaired lactation, and increased maternal anxiety. Interestingly, F2 dams also exhibit depressed maternal care and elevated maternal anxiety (Nephew, unpublished data). OXT and GR gene expression in both HPA and maternal care and reward associated regions are altered in F0 and F1 dams. Together with epigenetic modifications, these are associated with maladaptive changes possibly underpinning maternal care and anxiety [46–48].

A further emerging concept in the behavioral epigenetics field is that stress prenatally, or even preconceptionally, could produce alterations in behavior in offspring and grand-offspring (F1–F2 generations) through epigenetic changes via the germline. The term epigenetics refers to heritable changes in gene expression that does not involve changes to the underlying DNA sequence; three systems that can interact with each other to silence or activate genes are DNA methylation, histone modifications, and RNA-associated silencing. Epigenetics may mediate transmission of behavioral phenotypes across generations through interactions with early life care. That such epigenetic marks might also be directly transmitted through the germline is an active area of research [112]. Results from the CSS model suggest that potential epigenetic mechanisms underlying predisposition/transmission, through environmental stressors of social conflict and impaired maternal care, may be a key factor in the transgenerational accumulation of environmental insults and more relevant to the majority of individuals that suffer from mild to moderate depression that is more likely to involve complex environmental interactions.

In the first study in the literature to show a clear link between mothering, long-term changes in epigenetic marks (DNA methylation), and subsequent gene expression, Weaver et al. demonstrated that in rat pups of low licking and grooming (LG) mothers NR3C1 (the gene for GR) methylation was increased in hippocampal samples and that this was associated with reduced GR expression [113]. Very few studies have identified candidates for human maternal behaviors equivalent to rat LG, however, a study by Sharp et al. demonstrated moderation of the effects of prenatal maternal depression upon emotional and physiological outcomes in human infants through mothers stroking their babies in their first weeks of life [114]. A very recent follow-up study by Murgatroyd et al. has shown reduced NR3C1 methylation associated with maternal stroking in these children, hence bolstering the possible role of epigenetic mechanisms in the long-term effects of early life stress and maternal care [115]. Interestingly, the same study also found interactive effects between prenatal and postnatal maternal depression on methylation of NR3C1. Infants of mothers with low prenatal depression showed increased methylation when exposed to increased postnatal depression - consistent with interplay between prenatal and postnatal environments.

While most of the animal studies of the consequences of early social environment on neurophysiology and behavior have focused on the HPA axis and GR regulation, it would be advantageous to increase use of these models to investigate the influence of early life stress on social and reward pathways to fully understand the transgenerational transmission of depression. Ethologically relevant cross domain investigations of the reward system may lead to substantial progress in preventing the transmission of depression from mothers to offspring [116]. In two such studies, sensitizing adult female rats to cocaine (only during the adult phase) increased the expression of maternal care when those rats were later exposed to foster pups [117] or caring for their own young [118]. The increase in maternal care was associated with changes in neural activity in the anterior thalamus and periaqueductal gray of foster pup exposed nulliparous females [117], and it is postulated that the enhanced maternal care in the cocaine experienced nulliparous and primiparous dams is due to cross sensitization between cocaine and maternal care. These studies suggest that priming the reward pathway, possibly with stimuli other than cocaine, may be an effective strategy to prevent impaired maternal care in women at risk for PND, such as those with a history of depression or anxiety.

## Maternal Reward Deficits in Drug Use Disorders

Acute intake of a drug, such as cocaine, affects maternal behavior due to its direct molecular actions affecting the activity of neurons within the reward pathway, primarily its effects on mesolimbic and mesocortical dopamine (DA). The acute and chronic effects of cocaine have been studied in detail in rodent models. There are dose-dependent disruptive effects of cocaine on maternal behaviors [119]. Once plasma levels of cocaine are reduced, normal maternal care resumes [120, 121]. Thus, the acute effects of cocaine in drug naïve maternal rats may be reversible at the doses previously tested [120, 121]. Behavioral features of cocaine use disorders in humans are best captured by the rat intravenous drug self-administration paradigm, especially if it incorporates long-term reinstatement of drug intake [122]. Using the former model of cocaine intake it was shown that rats trained to administer cocaine prior to pregnancy escalate intake during pregnancy and reduce their consumption during the postpartum period [123]. Thus, during the postpartum period there is a reduction in drug self-administration in rats, likely associated with a greater focus on behaviors directed at nest building, nursing and pup grooming. It is difficult to reconcile the latter findings in rats with reports in humans since there are well-reported effects of drug use disorders on maternal care [124–126]. However, it is interesting to note that rat models of escalated intake with 6 h of daily self-administration sessions [127] have provided additional insight on the effects of cocaine on DA dynamics that are distinct from those of 2 h self-administration paradigms. Escalated (6 h) but not standard 2 h sessions of cocaine IVSA reduce transient bursts of DA release in striatum [128], which could have an effect on maternal care as this transient phasic DA release is modulated during the expression of grooming pups and nursing, as well as during social interactions [129–131]. It remains to be determined if escalated cocaine intake could have such an effect on brain reward DA pathway activation and maternal behavior in rats.

As discussed above, studies using rat models of maternal behavior support the notion that newborn pups are highly rewarding in lactating rats. There is behavioral and animal neuroimaging support suggesting that the rewarding effect produced by pups is greater than that produced by cocaine [8, 9, 123, 132, 133]. However, in the case of mothers with prior history of a drug use disorder, it is possible that the neural mechanisms underlying such rewarding effects may be adversely affected. Mothers affected by a cocaine use disorder may experience challenges to their roles as care givers. This is supported by data showing reduced mother-child play interactions, having a low self-esteem and lack of maternal identity, not attending to the emotional needs of the child and difficulties in coping with stress [125, 134, 135]. There is also evidence from the National Survey on Drug Use and Health that prior history of repeated drug use before pregnancy may be related to resuming high levels of intake during the postnatal care period (this statistically significant pattern is mostly observed for marihuana, cigarettes and alcohol misuse)(2009). Problems with parenting behavior in mothers recovering from a drug use disorder have been associated with negative early childhood experiences [136], which hints at the possible impact of early life adverse experiences on future expression of maternal behavior and attachment. In addition to the socioeconomic factors that clearly are important, there may also be long-term neurobiological changes in the reward system following chronic drug use that could influence maternal care during the early postnatal period.

Similar to OXT, normal DA function may be critical to the expression of maternal behavior [30]. At the moment of the present review there have been no studies that have used positron emission tomography to measure *in vivo* DA receptor levels or DA release in mothers affected by a drug use disorder. Such clinical studies would provide direct evidence of DA alterations in mothers diagnosed with a drug use disorder. Thus, most data on DAergic changes with drug exposure have come from studies using rodent models [137]. Maternal rats show elevated extracellular DA in NAc in the presence of pups [4, 130]. It appears that DA dynamics in NAc are closely associated with specific maternal-offspring interactions, such as grooming and nursing [4, 130]. Neurotoxin-mediated destruction of DA inputs in NAc impairs pup retrieval behavior [138] as does blocking DA receptors with a non-selective antagonist (*Cis*-flupenthixol) [139]. Numan et al. [140] demonstrated that retrieval behavior is modulated by DA D1 receptors in the shell areas of the NAc. Given the importance of NAc DA in reinforcement and rewarding effects of natural rewards and drug stimuli, it is likely that higher DA levels contribute to maternal-offspring attachment through the enhancement of brain reward function. However, to confirm that this is in fact the case, studies applying techniques such as intracranial self-stimulation to examine changes in brain reward thresholds are needed. Nonetheless, blocking DA receptors either systemically or locally within this region results in a disruption of various maternal behaviors such as retrieval, grooming, nest building and hovering over pups [139, 141, 142]. These results demonstrate that DA deficits negatively impact maternal behaviors in the rat and open the possibility that functional deficits in DA pathways could adversely affect maternal care in humans as well.

In addition to DA in NAc, PFC DA might also play an important role in maternal behaviors and maternal-offspring attachment. Previous imaging studies in rats have shown that pup suckling stimulation activates areas of the PFC [9, 143]. One reported long-term effect of repeated pregestational cocaine in the maternal brain is a reduced neural response to pup suckling in comparison to non-exposed dams [144]. The PFC of maternal rats, like in non-maternal rats, may play a role in complex higher order reward-seeking and cognitive functions that are important for maternal behaviors and non-maternal social behaviors. Using a modified neuroimaging paradigm to present *in vivo* natural stimuli in an fMRI environment, Nephew et al. reported that several PFC subregions, including anterior cingulate (ACg), orbital cortex and insula, show increased neural activity in response to the presentation of a male intruder in the presence of pups [145, 146]. Inhibiting the medial PFC just ventral the ACg in maternal rats significantly impairs retrieval of pups to a nest region of their home cages [147] and neurons within this area are responsive during maternal-offspring interactions [148]. A role for PFC in maternal care and its potential role in deficits in maternal reward is not unexpected given its widespread corticocortical connectivity and its extensive outputs to striatal and other limbic targets involved in maternal behavior [149–155]. Synaptic targets of various prefrontal areas and mPFC include subregions of the hypothalamus such as the medial preoptic area, VTA, periaqueductal grey (PAG) and amygdala [153, 154]. The ventral and dorsal striatum, VTA, basolateral amygdala, septum and PAG, for example, receive direct mPFC synaptic inputs [154]. In addition, the mPFC receives inputs from VTA DA neurons [156]. While the release of DA in the maternal NAc has been investigated, the role of DA activity in PFC of maternal rats needs further investigation in relation to drug misuse and co-morbid conditions such as generalized anxiety, obsessive-intrusive thoughts, PPD and other recurrent major depressive disorders.

There are still open questions about potential parent-to-offspring transmission of epigenetic factors that could lead to vulnerability to deficits in DA reward pathway function. Studies of human maternal behavior and drug consumption and its effects on children focus on the offspring effects of maternal consumption of a number of illicit and legal drugs, including cocaine, amphetamine, marihuana and nicotine [157–159]. Attachment seems to be negatively affected by drug use [126]. However, a significant number of studies focus on pregnancy use. Given the teratogenic actions of many of these drugs it is likely that the outcomes on brain development during the postnatal period may be a combination of drug-mediated effects and postnatal care effects. The outcomes of *in utero* exposure to drugs such as cocaine have been extensively reported for rodent models [100, 160]. Thus, it has been difficult to disentangle the separate effects of drug exposure *in utero* from behavioral disorders arising from lack of appropriate postnatal parental care. One of the best pieces of evidence of transgenerational effects are retrospective studies of mothers with a drug use disorder and that have difficulties in caring for their children [161–163]. In many of these assessments it has become clear that early child abuse and neglect, chronic stress, and parental drug use have had a long-term impact on the present behavior of the mother [100, 136, 162–164]. The early life experiences may lead to long-term alterations in OXT and DA systems, thereby impacting the social, cognitive, emotional behaviors modulated by these neurotransmitter systems [30]. Moreover, negative early life experiences, such as the stress induced in newborn pups by maternal separation, differentially alter the expression of D1 and D2 DA receptors in PFC-NAc excitatory projections during adolescence [165].

## Conclusion

There is substantial evidence that the reward pathway plays a key role in maternal responding to infant cues. This is supported by an extensive literature in rodent models of maternal behavior and also human neuroimaging and psychosocial/clinical research. Postpartum depression and drug use disorders may entail alterations in the reward pathway, particularly in striatal and prefrontal areas, which may affect maternal attachment to offspring and heighten the risk of transgenerational effects on the OXT and DA systems. Alterations may involve neural circuitry changes, genetic factors that impact monoaminergic neurotransmission, as well as growth factors such as BDNF and stress-associated signaling in the brain. Improved maternal reward-based preventative measures and treatments may be specifically effective for mothers and their offspring suffering from depression and/or addiction.
